# Developmental and angiogenic safety profiles of novel pyridine-based chalcone

**DOI:** 10.3389/fphar.2026.1780086

**Published:** 2026-04-17

**Authors:** Arij Fouzat Hassan, Hadeel Kheraldine, Sourour Idoudi, Ola J. Hussein, Hamda Al-Thawadi, Ashraf Khalil, Ala-Eddin Al Moustafa, Abdelbary Elhissi

**Affiliations:** 1 Pharmaceutical Sciences Department, College of Pharmacy, QU Health, Qatar University, Doha, Qatar; 2 College of Medicine, QU Health, Qatar University, Doha, Qatar; 3 College of Pharmacy, Dubai Medical University, Dubai, United Arab Emirates; 4 Oncology Department, McGill University, Montreal, QC, Canada; 5 ABS Research Review & Consultation, Montreal, QC, Canada

**Keywords:** angiogenesis, chalcones, chorioallantoic membrane (CAM), embryogenesis, VEGF

## Abstract

**Introduction:**

Pyridine chalcone derivatives are emerging anticancer candidates with potent activity against different types of cancer. However, their developmental and angiogenic safety profiles remain unexplored. In our lab, we recently generated a series of novel pyridine-based chalcone analogs; we reported that two of them, OH17 and OH25, are highly effective against different types of cancer cell models.

**Methods:**

We herein investigated the effects of these two compounds on early embryonic development and normal vascular formation using the chicken embryo and chorioallantoic membrane (CAM) models. In addition, qPCR analysis of key apoptosis- and angiogenesis-related markers was performed on organs collected from treated embryos, together with *in vitro* evaluation using normal embryonic fibroblast cells (NEFCs).

**Results:**

Our data revealed that both OH17 and OH25 did not affect the normal development of the embryos and their survival rate when they were exposed to these two compounds at 3 days of incubation. Meanwhile, the CAM analysis showed no significant alterations in vessel area, branching, or density, with only a minor reduction in average vessel length. More significantly, gene expression patterns of apoptotic and angiogenic key markers remain unchanged across different tissues from several organs of exposed embryos. Consistently, NEFCs retained normal viability and fibroblast morphology following treatment with the two compounds.

**Discussion:**

Together, these findings, combined with our previous reports, demonstrate OH17 and OH25 safety in addition to their potent anticancer activity. Thus, paving the way towards a favorable therapeutic window for these two compounds in support of their further development as promising anticancer agents.

## 1 Introduction

Chalcones represent a structurally simple yet biologically versatile class of compounds, widely recognized for their diverse pharmacological properties (Salehi et al., 2020). Characterized by an α,β-unsaturated carbonyl system linking two aromatic rings, chalcones serve as key intermediates in the biosynthesis of many natural products and have been extensively explored in medicinal, agricultural, and biochemical research (Villa et al., 2024; Kamya et al., 2021; Jasim et al., 2021). Their ease of synthesis and highly modifiable scaffold allow chemists to generate numerous derivatives with tailored biological attributes (Elkanzi et al., 2022). Among these, pyridine-based chalcones have attracted increasing interest due to the unique physicochemical properties conferred by the nitrogen heteroatom (Sankhe and Mukadam, 2024). While many chalcone derivatives have been investigated for antimicrobial (dos Santos et al., 2023), antioxidant (Zheng et al., 2025), anti-inflammatory (ur Rashid et al., 2019), and anticancer activities (Ouyang et al., 2021). Numerous chalcone derivatives have demonstrated promising anticancer outcomes against a broad spectrum of malignancies, including breast (Elkhalifa et al., 2020; Rizeq et al., 2021), colorectal (Hassan et al., 2024; Alaa et al., 2020), lung (Abdelaal et al., 2024), and prostate cancers (Hussein et al., 2025), through mechanisms such as apoptosis induction, cell cycle arrest, inhibition of angiogenesis, suppression of metastasis, and modulation of key oncogenic signaling pathways (Chowdhary et al., 2024).

Collectively, these findings highlight the strong therapeutic potential of chalcone-based compounds as anticancer agents. However, the advancement of chalcones toward clinical translation requires not only demonstration of efficacy, but also thorough evaluation of their biosafety profiles (Salehi et al., 2020; Sahu et al., 2012). To this end, various *in-vivo* models, including rodents and zebrafish embryos, have been widely employed to assess the toxicity of chalcone derivatives. Despite these efforts, significantly less is known about their developmental and angiogenic effects, particularly during the early stages of vertebrate growth (Lee et al., 2014; Jie et al., 2020). Embryonic development is a highly regulated and vulnerable process in which precise cellular signaling, tissue differentiation, and organogenesis occur in a coordinated sequence (Ellis-Hutchings et al., 2017; Antonouli et al., 2025). Small molecules capable of modulating biological pathways in mature cells may exert different or unexpected effects during embryogenesis, where even subtle disruptions can interfere with viability or normal growth (Barrow and Clemann, 2021; Marikawa, 2022).

One of the most critical processes during early development is angiogenesis, the formation and expansion of new blood vessels (Kheraldine et al., 2024a). Angiogenesis supports essential embryonic functions, including oxygen delivery, nutrient transport, waste removal, and the establishment of physiological gradients required for morphogenesis (Trimm and Red-Horse, 2023; Huveneers and Phng, 2024). Disturbances in vascular growth can lead to developmental abnormalities, growth retardation, or embryonic lethality (Persson and Buschmann, 2011). Consequently, understanding how newly synthesized chalcone derivatives interact with the early stage of the embryo including its blood vessel development is an important component of assessing their broader biological safety.

The chicken embryo and its chorioallantoic membrane (CAM) model offers a powerful, ethically accessible, and highly visual platform for evaluating such developmental interactions (Mosna et al., 2025). The CAM is a naturally vascularized extraembryonic membrane that expands rapidly during mid-embryogenesis, making it exceptionally suitable for imaging blood vessel formation and assessing angiogenic disturbances (Ribatti, 2016; Ribatti et al., 2020). The model allows real-time observation, controlled compound application, and quantitative analysis of vascular parameters such as vessel density, branching, and morphology (Nowak-Sliwinska et al., 2014). In addition to its relevance for angiogenesis research, the chicken embryo model supports investigations into general embryotoxicity, tissue sensitivity, and early developmental responses to chemical exposure (Acharya et al., 2024).

On the other hand, our group recently generated a series of novel pyridine-based chalcone analogs; we showed that two of them, OH17 and OH25, are highly effective against prostate and triple negative breast cancer cell models (Hussein et al., manuscript under review) and (Hassan et al., manuscript under review). Thus, we herein explored the safe outcome of OH17 and OH25 on the early stages of development and angiogenesis using the chicken embryo and its CAM model. We also used primary embryonic fibroblast cells (NEFCs) derived from chicken embryos as an *in vitro* system for assessing cellular and molecular responses in normal, non-specialized embryonic tissue (Mahmoud et al., 2021). These compounds were selected based on their documented biological activity in previous studies, and a single test concentration of 2 µM was chosen to provide a meaningful and biologically relevant exposure level. We herein present a comprehensive analysis related to angiogenesis, embryo survival, morphological evaluation and gene expression patterns. Thereby, this study provides a broad and detailed perspective on the potential embryotoxic, angiogenic, and cellular interaction of the two pyridine chalcone compounds.

## 2 Materials and methods

### 2.1 Drugs and reagent

The pyridine chalcone derivatives OH17 and OH25 were synthesized via a Claisen–Schmidt condensation reaction and purified in accordance with the procedure reported in our published patent (US 2025/0170111 A1) (Ashraf et al., 2025). Each compound was initially dissolved in dimethyl sulfoxide (DMSO) to obtain a 5 mM stock solution. For experimental use, the stock solution was diluted to a final concentration of 2 µM. All chalcone solutions were stored at −20 °C, protected from light.

### 2.2 Chicken embryos

Fertilized White Leghorn chicken eggs (MAZZRATY - National Group for Agriculture and Animal Products, Qatar)/Arab Qatari for Poultry Production, Qatar) were incubated at 37 °C and 60% relative humidity using a MultiQuip incubator. To prevent adhesion of the embryo to the surrounding membranes, the eggs were rotated every hour. Embryos at three and 5 days of incubation were used for the embryogenesis and angiogenesis assessments, respectively. The embryos were treated with the chalcone compounds OH17 and OH25 at a final concentration of 2 μM, and responses were compared to control embryos receiving DMSO alone, following protocols routinely used by our group (Kheraldine et al., 2024a; Kheraldine et al., 2026). For the angiogenesis study, images were captured 48 h after treatment and analyzed with AngioTool Software (version 0.6a) (Zudaire et al., 2011). For the evaluation of embryogenesis, embryo autopsy was performed 5 days after treatment, and organs including the heart, brain and lungs were harvested for RNA extraction and subsequent qPCR analysis. The concentration of 2 µM was selected based on our previous findings demonstrating that both OH17 and OH25 exhibit half-maximal inhibitory concentrations (IC_50_) of approximately 2 µM in triple-negative breast cancer (TNBC) cell lines (Hassan et al. unpublished data). This dose therefore represents a biologically relevant concentration associated with anticancer activity. All experimental procedures involving chicken embryos were reviewed and approved by the Institutional Bio-Safety Committee (IBC) of Qatar University under protocol number QU-IBC-058/2024-AMM2 and by the Institutional Animal Care & Use Committee (QU-IACUC) of Qatar University under approval number QU-IACUC-008/2025-AMM1. All procedures were conducted in accordance with institutional and national guidelines for the ethical use of animals in research.

### 2.3 RNA extraction and qPCR

Total RNA was isolated from the collected embryonic tissues (brain, heart, and lungs) using the NucleoSpin TriPrep Mini kit (MACHEREY-NAGEL, Germany) following the manufacturer’s protocol. RNA concentration was determined using a NanoDrop spectrophotometer (Thermo Fisher Scientific, United States), and purity was assessed by the 260/280 absorbance ratio, with values near 2.0 indicating high-quality RNA. Complementary DNA (cDNA) was then synthesized using the SuperScript™ III First-Strand Synthesis SuperMix kit (Thermo Fisher, United States) according to the supplier’s instructions. Gene expression analysis was performed by quantitative real-time PCR (qPCR) using the iTaq™ Universal SYBR® Green Supermix (BIO-RAD, Australia). The qPCR assays evaluated the expression of key apoptosis- and angiogenesis-related genes, including caspase-3, caspase-8, caspase-9, Bax, and VEGF, with glyceraldehyde-3-phosphate dehydrogenase (GAPDH) used as the endogenous reference gene. Primer sequences are listed in Table 1. Amplification reactions were run on a QuantStudio® 5 Real-Time PCR System.

**TABLE 1:** Primer sequences used for qPCR analysis.

Primer	Forward primer (5′–3′)	Reverse primer (5′–3′)
Caspase-3	F: 5′- GAT​CAG​GAC​GAG​CAG​GAC​G -3′	R: 5′- TGT​CCA​GAT​GCC​ACA​GTT​C -3′
Caspase-8	F: 5′- TGA​GTA​CGC​TGT​TTG​CTC​TG -3′	R: 5′- CTT​GAC​GTT​GGG​TTG​ACT​TG -3′
Caspase-9	F: 5′- CAG​CGT​GTT​TGT​CCT​GAC​TG -3′	R: 5′- TCT​GAG​GCG​TGA​CCT​GAG​TG -3′
BAX	F: 5′-TCA​CAG​CCA​GGA​GAA​TCG​CAC -3′	R: 5′-GCT​GCA​GAC​ATG​CTG​TGG​ATC -3′
VEGF	F: 5′- AGC​CTC​CTC​CTG​GTG​CTT​CT -3′	R: 5′- TGT​GAT​GAT​TGC​TGC​TTG​TG -3′
GAPDH	F: 5′- GAA​GGT​GAA​GGT​CGG​AGT​C -3′	R: 5′- GAA​GAT​GGT​GAT​GGG​ATT​TC -3′

### 2.4 Cell culture

The effects of the chalcone compounds OH17 and OH25 were further assessed using embryonic fibroblasts (NEFCs) derived from 9-day-old chicken embryos. The fibroblast culture was established in our laboratory using standard isolation procedures (Kheraldine et al., 2024a). Briefly, embryos were removed from their eggs, after which the limbs and internal organs were carefully dissected. The remaining tissues were subjected to repeated digestion with 0.25% trypsin–EDTA containing phenol red (Gibco, Life Technologies, United States) to obtain a single-cell suspension. The resulting cells were cultured in complete growth medium consisting of RPMI-1640 (Gibco, Life Technologies, United States) supplemented with 10% fetal bovine serum (FBS) and 1% Pen–Strep (Invitrogen, Life Technologies, United States). Cells were maintained at 37 °C in a humidified incubator with 5% CO_2_. All experiments were performed when cultures reached approximately 70%–80% confluence.

### 2.5 Cell viability assay

Cell viability was evaluated using the Alamar Blue assay following standard protocols. NEFCs were seeded into 96-well plates at a density of 5 × 10^3^ cells per well and allowed to adhere overnight. Once cells reached approximately 70%–80% confluence, they were treated with the chalcone compounds OH17 and OH25 at different concentrations, while control wells received an equivalent volume of DMSO. After 48 h of incubation, 2% (v/v) Alamar Blue reagent (Invitrogen, Life Technologies, United States) was added to each well, and the plates were returned to the incubator for 3 h. Fluorescence intensity was then measured using a microplate reader at an excitation wavelength of 560 nm and emission at 590 nm. Cell viability was calculated as a percentage relative to the DMSO-treated control group.

### 2.6 Morphological examination

Morphological changes in NEFCs following treatment with OH17 and OH25 were assessed using phase-contrast microscopy. Cells were seeded in 6-well plates and allowed to reach 70%–80% confluence. Cells were then treated with 2 µM of either OH17 or OH25, while control wells received DMSO alone. After 48 h of incubation, cells were examined using DMi8 inverted microscope (Leica, Germany) at ×10 magnification. Representative images were captured using the integrated digital imaging system.

### 2.7 Statistical analysis

All data are presented as mean ± standard error of the mean (SEM). Statistical analyses were performed using GraphPad Prism software (version 10; GraphPad Software, United States). Differences among treatment groups were assessed using one-way analysis of variance (ANOVA) followed by Tukey’s *post hoc* test for multiple comparisons. For qPCR data, relative gene expression was calculated using the 2^(−ΔΔCt) method after normalization to GAPDH. Embryo survival was analyzed using Kaplan–Meier survival curves, and statistical differences between groups were assessed using the Holm–Sidak multiple comparison test. A *p-value* of less than 0.05 was considered statistically significant. All experiments were performed in triplicate unless otherwise specified.

## 3 Results

In this study, 3 day-old chicken embryos were treated with 2 µM of the chalcone derivatives OH17 or OH25 consistent with our recent findings (Hassan et al., manuscript under review) for 5 days to evaluate their effect on the early embryonic development. Embryo survival was monitored over a 5 day period following treatment with OH17 or OH25. Embryos were treated with 2 µM of both compounds, a concentration selected to match the IC_50_ previously established in our cytotoxicity assays (Hassan et al., manuscript under review) ensuring that the developmental evaluation was conducted at a therapeutically meaningful dose.

**FIGURE 1:**
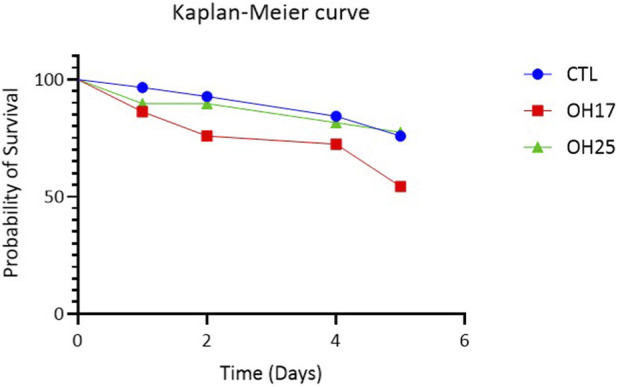
Survival rate of chicken embryos treated with chalcone derivatives OH17 and OH25. Kaplan–Meier survival curve comparing the probability of survival of chicken embryos treated with 2 µM of OH17 or OH25 relative to the control over a 5-day period post-treatment. Both compounds displayed survival profiles comparable to the control group, with OH25 closely matching control viability and OH17 showing a modest, non-significant decline in survival. Statistical comparison of survival distributions was performed using the Holm–Sidak multiple comparison test.

**FIGURE 2:**
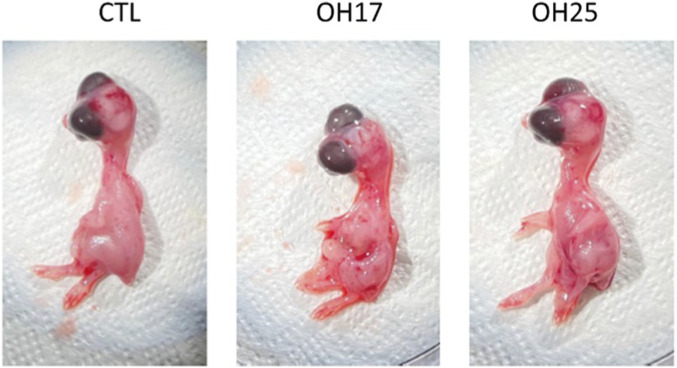
Morphological assessment of chicken embryos following treatment with chalcone derivatives OH17 and OH25. Representative images of chicken embryos collected 5 days after treatment with 2 µM OH17, or 2 µM OH25. Embryos across all groups exhibited normal gross morphology, with no observable signs of developmental delay, hemorrhage, malformations, or structural abnormalities. Overall body shape, limb formation, and organ appearance remained consistent with typical embryonic development at this stage, indicating that treatment with OH17 or OH25 did not induce overt embryotoxic effects.

According to the Kaplan–Meier survival curves, neither compound caused a statistically significant reduction in embryo viability when compared with the control group (Holm–Sidak *post hoc* test; *p > 0.05*) (Figure 1). Although a gradual decline in survival was observed across all groups as incubation progressed, the control and OH25-treated embryos displayed similar survival trajectories, maintaining approximately 80% viability by day 5. In contrast, embryos treated with OH17 showed a slightly greater decline in survival, reaching approximately 55% by day 5; however, this reduction did not reach statistical significance. Additionally, embryo morphology and developmental progression remained normal across all treated groups. As shown in Figure 2, no evidence of growth delay, hemorrhage, malformations, or impaired embryogenesis was observed, confirming that exposure to OH17 and OH25 does not adversely affect the early development. Overall, these results demonstrate that exposure to our novel chalcone compounds did not significantly impair embryo survival during early development, supporting a generally low embryotoxic profile for our chalcone derivatives.

In concordance with our recently published work on the effect of similar compounds on embryogenesis (Rizeq et al., 2021; Kheraldine et al., 2024a; Kheraldine et al., 2024b), and to determine the effect of OH17 or OH25 on brain, heart and lung tissues. The expression levels of key apoptosis-associated genes (caspase-3, caspase-8, caspase-9, and Bax) as well as the angiogenic marker VEGF were evaluated and normalized to GAPDH, using quantitative real-time PCR. Thereby, mapping possible molecular alterations related to apoptosis or angiogenesis during the early development of these organs.

**FIGURE 3:**
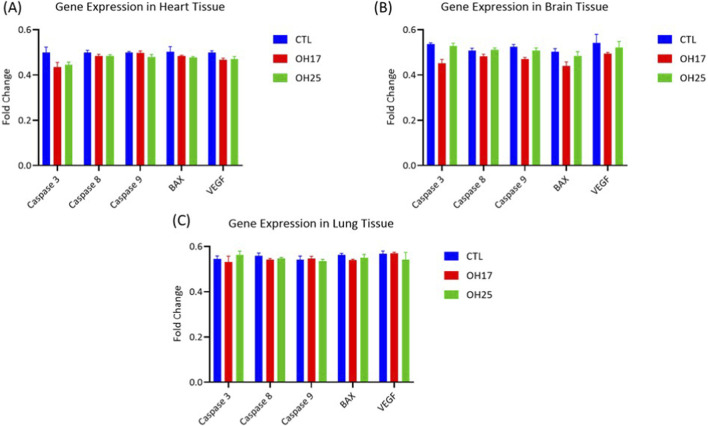
Quantitative real-time PCR analysis of apoptosis- and angiogenesis-related gene expression in embryonic tissues following treatment with OH17 and OH25. Relative mRNA expression levels of caspase-3, caspase-8, caspase-9, Bax, and VEGF were assessed in heart **(A)**, brain **(B)**, and lung **(C)** tissues collected from embryos treated with 2 µM OH17 or OH25 and compared with control. Gene expression remained unchanged across all groups, with no significant upregulation or downregulation detected for any of the examined markers. Data are presented as percentage expression relative to control (mean ± SEM, n = 3). Statistical analysis was performed using one-way ANOVA followed by Tukey’s *post hoc* test. No statistically significant differences were observed (*p > 0.05*). VEGF: Vascular endothelial growth factor; Bax: Bcl-2–associated X protein.

As shown in Figure 3, none of the examined genes exhibited significant changes in expression following treatment with either chalcone derivative. In all three tissues analyzed, mRNA levels of caspase-3, caspase-8, caspase-9, Bax, and VEGF remained comparable to those of the control group (*p > 0.05*). This uniform pattern across multiple organs indicates that OH17 and OH25 did not activate apoptotic pathways nor suppress pro-angiogenic signaling at the transcriptional level. These findings suggest that, at the tested concentration of 2 μM, both chalcone derivatives exert minimal molecular impact on early embryonic development, confirming the overall safety profile observed in the angiogenesis and survival assays.

Next, 5-day-old chicken embryos were treated with both chalcone derivatives at the same concentration for 48 h to evaluate their effects on angiogenesis. The treatment was applied directly onto the chorioallantoic membrane (CAM) following the established windowing protocol used routinely in our laboratory, enabling clear visualization and accurate comparison between treated and untreated regions of the CAM (Kheraldine et al., 2024b).

**FIGURE 4:**
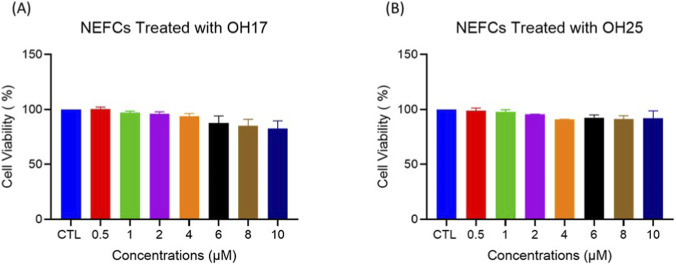
Angiogenesis assay of the CAM in chicken embryos. The embryos were treated with 2 µM of both OH17 and OH25. **(A)** Representative stereomicroscopic images of the CAM showing the vascular architecture 48 h after treatment with OH17 (2 µM), or OH25 (2 µM) compared to the control. Both compounds preserved the overall vascular network structure, with no visible changes in vessel density, branching, or capillary formation compared to the control group (n = 3). **(B)** Quantification of angiogenesis parameters, including vessel area, vessel percentage area, total vessel length, number of junctions, number of endpoints, and average vessel length. OH17 and OH25 did not significantly alter any of the measured vascular parameters except for a reduction in average vessel length, which was significantly decreased compared to the control. Data are presented as mean ± SEM (n = 3). Statistical analysis was performed using one-way ANOVA followed by Tukey’s *post hoc* test. Results were considered statistically significant when *p-value ≤ 0.05*. *p ≤ 0.05 and **p ≤ 0.01.

As shown in Figure 4A, visual inspection of the CAM revealed that both OH17- and OH25-treated embryos did not produce visible alterations in the overall vascular architecture of the CAM when compared with the control. Embryos in all groups displayed a well-organized vascular network with intact primary and secondary vessels, and no gross differences were observed in branching pattern, vessel density, or capillary formation. Quantitative vascular analysis using AngioTool further supported these observations (Figure 4B). Neither OH17 nor OH25 caused significant changes in vessel area, vessel percentage area, total vessel length, total number of junctions, or number of endpoints relative to controls (Figure 4B; *p > 0.05*). Despite this preserved global angiogenic profile, both compounds induced a significant reduction in average vessel length compared to the control group (*p < 0.05*), indicating a subtle yet measurable effect on vascular morphology.

In parallel, primary embryonic fibroblasts (NEFCs) were successfully generated from 9-day-old chicken embryos and cultured under standard growth conditions, as described in the Materials and methods section.

**FIGURE 5:**
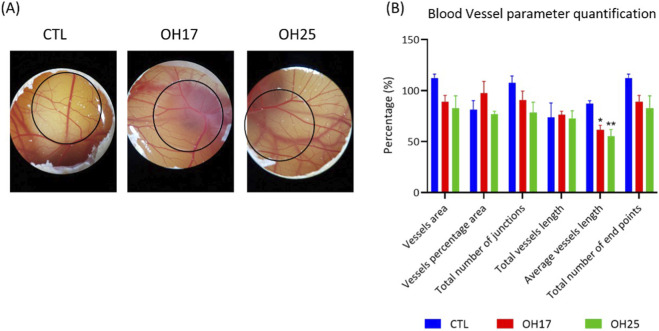
Viability of primary embryonic fibroblasts (NEFCs) following treatment with chalcone derivatives OH17 and OH25. Primary NEFCs isolated from 9 day-old chicken embryos were treated with OH17 or OH25 for 48 h, and cell viability was assessed using the Alamar Blue assay. **(A)** and **(B)** Representative bar graphs showing cell viability across experimental groups. Both compounds-maintained fibroblast viability at levels comparable to the control, with no statistically significant reduction observed (*p > 0.05*). Data are presented as mean ± SEM (n = 3). Statistical analysis was performed using one-way ANOVA followed by Tukey’s *post hoc* test.

To evaluate whether exposure to chalcone derivatives affects the viability of normal embryonic cells, NEFCs were treated with a different dose of OH17 or OH25 (0.5,1,2,4,6,8,10 µM) for 48 h, and cell viability was assessed using the Alamar Blue assay. As shown in Figure 5, neither OH17 nor OH25 induced a remarkable reduction in cell viability when compared with the control group. Cell viability remained above 90% across all treatment groups, and no statistically significant differences were observed (*p > 0.05*). These findings indicate that both compounds exhibit minimal cytotoxicity toward embryonic fibroblasts at the tested concentrations, supporting the overall safety profile demonstrated in the *in-ovo* assays.

**FIGURE 6:**
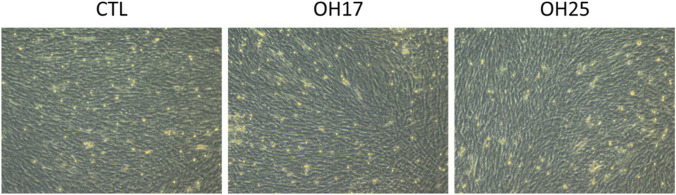
Morphological assessment of primary embryonic fibroblasts (NEFCs) following treatment with chalcone derivatives OH17 and OH25. Representative phase-contrast microscopy images of NEFCs after 48 h exposure to 2 µM of OH17 or OH25. Cells in all groups displayed normal fibroblast morphology, characterized by elongated spindle-shaped structures, intact cell membranes, and uniform monolayer organization. No signs of cytotoxicity such as rounding, shrinkage, detachment, or loss of adherence were observed in treated cells compared to controls. Images were captured at ×10 magnification.

To further evaluate the cytotoxic potential of OH17 and OH25 on normal embryonic cells, the morphology of the NEFCs was examined 48 h after treatment. Phase-contrast microscopy revealed that NEFCs in all groups control, OH17-treated, and OH25-treated, displayed the typical elongated and spindle-shaped morphology characteristic of healthy fibroblasts (Figure 6). Cells remained well-attached, maintained uniform orientation and confluence, and showed no signs of membrane damage, rounding, shrinkage, or detachment. Consistent with the viability assay findings, neither compound induced observable morphological abnormalities nor structural changes in the fibroblast monolayer. These results indicate that OH17 and OH25 exert no detectable morphological toxicity on embryonic fibroblasts at the tested concentration of 2 μM, further supporting their minimal cytotoxic impact on embryonic cells.

## 4 Discussion

In this study, we performed a comprehensive evaluation of two novel pyridine chalcone derivatives, OH17 and OH25, to determine their potential effects on the early stage of the embryo and angiogenesis using the chicken embryo model and its CAM. We also used normal embryonic fibroblast cells (NEFCs) isolated from the embryo to confirm the cytotoxicity of the compounds on these cells. OH17 and OH25 were previously generated and reported by our group to exhibit potent anticancer activity with IC_50_ values around 2 µM (Hassan et al., manuscript under review). Therefore, this biologically relevant concentration was selected to assess whether the therapeutic window that produces cytotoxicity in cancer cells also carries developmental or vascular risks *in ovo*. Our findings demonstrate that, despite their anticancer potency, both chalcones exhibit a favorable safety profile in the chicken embryo and do not induce significant developmental, vascular, or molecular toxicity.

Based on our results, embryo survival and morphology remained largely unaffected by OH17 and OH25. This contrasts with previous studies demonstrating that some chalcones can induce embryotoxicity in zebrafish (Lee et al., 2014; Mohd et al., 2020). For instance, Lee et al. reported that zebrafish embryos that were exposed to a chalcone compound 1b exhibited multiple muscle abnormalities, characterized by disrupted and collapsed myofibrils, decreased cellular density, and disorganization of filament structures (Lee et al., 2014). In our study, however, neither compound compromised embryo viability nor caused observable malformations (Figures 2, 3). In fact, the chicken embryo CAM system is known to be more resistant than zebrafish but offers superior visualization of vascular development, making it an ideal model for assessing subtle angiogenic effects (Nowak-Sliwinska et al., 2014; Beedie et al., 2017).

At the molecular level, no significant changes were observed in apoptosis-related genes (caspase-3, caspase-8, caspase-9, Bax), indicating a lack of pro-apoptotic signaling in embryonic tissues (Figure 3). This contrasts with numerous cancer studies where chalcones robustly induce apoptosis through activation of caspase cascades and mitochondrial pathways (Ramirez-Tagle et al., 2016; Dong et al., 2018). For example, Zhu et al. demonstrated that chalcones strongly upregulate Bax and downregulate Bcl-2 in cancer cells, but have minimal effects on normal cells, supportive of what we observed in embryonic fibroblasts (Zhu et al., 2019). Our findings therefore reinforce the emerging concept that chalcones may preferentially target malignant cells while sparing normal tissues, a key characteristic for drug development.

Angiogenesis plays a central role in both embryonic development and tumor progression, and chalcones have been widely studied for their anti-angiogenic properties (Dudley and Griffioen, 2023; Burmaoglu et al., 2023). Interestingly, despite the known bioactivity of pyridine-based chalcones in cancer models, neither OH17 nor OH25 produced overt angiogenic disruption in the CAM (Figure 4). Quantitative image analysis in Figure 4B confirmed that key vascular parameters including vessel area, branching, endpoints, and total vessel length remained unchanged across all groups. The only parameter affected was the average vessel length, which showed a modest but statistically significant reduction. The absence of major angiogenic disruption observed in the CAM contrasts with several previous studies reporting strong anti-angiogenic effects of other chalcone derivatives (Senrung et al., 2023; Sun et al., 2021; Michalkova et al., 2021). For instance, Varinská et al. reported that a 4-hydroxychalcone (Q797) markedly suppressed angiogenesis by modulating both VEGF- and FGF-mediated angiogenic pathways and inhibiting endothelial cell functions *in-vivo* (Varinska et al., 2012).

Additionally, the modest reduction in average vessel length may reflect a subtle modulation of vascular elongation dynamics rather than a global inhibition of angiogenesis. Average vessel length is influenced by endothelial cell proliferation, migration, and sprouting behavior during active vascular remodeling. A slight decrease in this parameter, in the absence of changes in vessel density, branching, or total vascular area, suggests minor adjustments in vessel extension without disruption of overall vascular network architecture (Camasao and Mantovani, 2021). Biologically, this finding may indicate limited endothelial responsiveness to chalcone exposure that does not translate into functional impairment of angiogenesis (Ungvari et al., 2018). Such selective modulation, while preserving global vascular integrity, supports the conclusion that the compounds do not exert overt anti-angiogenic or developmental toxicity under the tested conditions.

Similar, several chalcone derivatives were shown to markedly reduce capillary density in the CAM assay. Supporting these findings, RT-qPCR analysis revealed that the anti-angiogenic activity correlated with altered VEGFR2 expression (Burmaoglu et al., 2023). Although these reports suggest chalcones can act as anti-angiogenic agents, the effect appears to be highly dependent on structural features and substitution patterns (George et al., 2022; Moreira et al., 2023). This minor vessel change, while the rest of the vasculature remains normal, suggests the compounds affect small structural details but not overall angiogenesis. Such a subtle effect may reflect early endothelial responsiveness to chalcone exposure, yet not to an extent that disrupts global vascular patterning.

Importantly, the lack of pronounced anti-angiogenic activity observed in the present study does not contradict the previously demonstrated anticancer potential of these chalcone derivatives. While angiogenesis is recognized as a hallmark of cancer progression, anticancer efficacy can arise through multiple tumor-cell intrinsic mechanisms independent of direct vascular inhibition (Lopes-Coelho et al., 2021). Several anticancer agents exert their effects primarily through induction of apoptosis, modulation of oxidative stress, disruption of cell cycle progression, or interference with oncogenic signaling pathways, without necessarily impairing physiological angiogenesis (Seaman et al., 2026; Gu et al., 2013; Nugitrangson et al., 2016). In this context, the preserved vascular architecture in the CAM model supports a favorable safety profile, suggesting that OH17 and OH25 may exert cytotoxic effects selectively on tumor cells while sparing normal developmental angiogenic processes. Such selectivity is therapeutically advantageous, as effective anticancer compounds ideally suppress malignant cell growth without compromising normal vascular function.

The preserved VEGF mRNA levels in our embryo tissues further support the minimal angiogenic impact of OH17 and OH25. Conversely, many chalcone derivatives show anticancer and anti-angiogenic activity, often mediated via suppression of angiogenic factors including VEGF in tumor or endothelial models (Adhikari et al., 2025; Silva et al., 2022; Barlak et al., 2025). However, certain chalcone analogues have been shown to exert anticancer activity without affecting VEGF gene expression *in-vitro* and *in-vivo* (Onda et al., 2015; Chen et al., 2015; Mirossay et al., 2017). Thus, our findings emphasize that not all chalcones share a universal anti-angiogenic mechanism; rather, their biological activity varies significantly across chemical families and biological systems.

Moreover, the viability and morphology of primary embryonic fibroblasts (NEFCs) further validate the low toxicity profile of OH17 and OH25 in non-cancerous cells. This is consistent with an earlier study demonstrating that trans-chalcone protects 3T3 embryonic fibroblasts from arsenic-induced toxicity (Unsal et al., 2024). In addition, dietary chalcone cardamonin (CD) enhanced apoptosis-driven cytotoxicity in tumor cells, while fibroblasts showed minimal loss of viability at the same treatment concentrations (Berning et al., 2019). Another study reported that trans-chalcone significantly affected the viability of breast cancer cells by inducing apoptosis with minimal effect on the 3T3 mouse fibroblast cell line (Bortolotto et al., 2017). These reports support our use of 2 μM, corresponding to the IC_50_ in cancer cells, and reinforce that a dose sufficient to compromise cancer cell viability does not elicit detrimental effects in healthy embryonic fibroblasts (Hassan et al., manuscript under review). Overall, our findings highlight a clear distinction between the anticancer potency and developmental safety of OH17 and OH25. While many chalcones exhibit broad anti-angiogenic or embryotoxic effects across various models, our pyridine-based chalcones display a more selective profile, producing minimal developmental or vascular disruption at therapeutically relevant doses. This suggests a potentially favorable therapeutic window for further development of these compounds.

Despite the strengths of the present study, several limitations should be acknowledged. The in ovo experiments were conducted at a single concentration corresponding to the previously determined IC_50_ value, and dose–response relationships were not assessed. Molecular analyses were limited to gene expression without protein-level validation of angiogenic markers. In addition, developmental exposure was restricted to an early embryonic window, and long-term developmental outcomes were not evaluated. Future studies should therefore explore broader dose ranges, extended developmental time points, protein-level validation of key signaling pathways, and *in vivo* pharmacokinetic profiling. Moreover, direct comparison of pyridine-based chalcones with other chalcone subclasses may help elucidate structure–activity relationships underlying the differential biological responses observed across systems.

## 5 Conclusion

In summary, the pyridine chalcones OH17 and OH25 showed a favorable safety profile in the chicken embryo model. At the therapeutically relevant concentration of 2 μM, neither compound affected embryo survival, morphology, angiogenesis, nor the expression of key apoptotic and angiogenic genes. Primary embryonic fibroblasts also maintained normal viability and morphology following treatment. Together, these findings highlight the minimal toxicity of OH17 and OH25 toward normal embryonic tissues and vascular structures at therapeutically relevant concentrations. This work paves the way for the continued development of pyridine-based chalcones as promising anticancer agents with a potentially wide therapeutic window and encourages further preclinical and clinical investigations across additional developmental and pharmacological models.

## Data Availability

The original contributions presented in the study are included in the article/supplementary material, further inquiries can be directed to the corresponding authors.
