# Correction: Developmental and angiogenic safety profiles of novel pyridine-based chalcone

**DOI:** 10.3389/fphar.2026.1862423

**Published:** 2026-05-07

**Authors:** Arij Fouzat Hassan, Hadeel Kheraldine, Sourour Idoudi, Ola J. Hussein, Hamda Al-Thawadi, Ashraf Khalil, Ala-Eddin Al Moustafa, Abdelbary Elhissi

**Affiliations:** 1 Pharmaceutical Sciences Department, College of Pharmacy, QU Health, Qatar University, Doha, Qatar; 2 College of Medicine, QU Health, Qatar University, Doha, Qatar; 3 College of Pharmacy, Dubai Medical University, Dubai, United Arab Emirates; 4 Oncology Department, McGill University, Montreal, QC, Canada; 5 ABS Research Review and Consultation, Montreal, QC, Canada

**Keywords:** angiogenesis, chalcones, chorioallantoic membrane (CAM), embryogenesis, VEGF

In the published article, the funding for the open access publication fees was omitted. The correct **Funding** statement: “The authors acknowledge the support of Qatar University for the Graduate Assistantship. Open Access funding for this article was provided by Qatar National Library (QNL), assisted by Qatar University (QU), and the Qatar Research, Development and Innovation Council (QRDI) linked to Grant No. NPRP9-337-3-069 (Principal Investigator: Professor A. Elhissi).

The original article has been updated.

